# Synthesis of Castor Oil-Based Quaternary Ammonium Salt and Modification of Attapulgite for Treating Industrial Wastewaters

**DOI:** 10.3390/ma16093468

**Published:** 2023-04-29

**Authors:** Xiuhua Yan, Jianfei Ding, Wenyan Shi, Lanqin Tang, Yidong Zhang, Wei Xu

**Affiliations:** School of Chemistry and Chemical Engineering, Yancheng Institute of Technology, Yancheng 224051, China; gyyxh@hotmail.com (X.Y.); jianfeiding@sina.com (J.D.); waterswy@126.com (W.S.); lanqin_tang@163.com (L.T.); zhyido@163.com (Y.Z.)

**Keywords:** castor oil, castor oil based-quaternary ammonium salt, removal rate, modified attapulgite, industrial wastewaters

## Abstract

In order to develop multifunctional quaternary ammonium salts and explore their advantages as modifiers for wastewater treatment, castor oil-based quaternary ammonium salts were synthesised and subsequently used as modifiers for attapulgite treatment. The structures of untreated and treated attapulgite were compared by Fourier transform infrared spectra and X-ray diffraction. The mechanism of modification was speculated. Various factors such as the amount of modified attapulgite, temperature and pH were also investigated in the batch experiments on the removal rates of acetone and phenol from wastewaters. The synthesis conditions were set as follows: the reaction temperature was 80 °C, the reaction time was 8 h, the molar ratio of castor oil to N,N-dimethyl-1,3-propanediamine was 1:5, the catalyst was 6% NaOH and the product yield was about 64.72%. The grafting rate of the castor oil-based quaternary ammonium salt was about 99.6% when the amount of modifier was 0.69 g per 5 g of attapulgite, the ultrasound treatment time was 11 min and the pH was 5. The quaternary ammonium salt was only associated with the surface of attapulgite and did not change the rod-like crystal structure of the silicate. The modified attapulgite is much more fibrous and exhibits a good distribution of crystal bundles. The removal rates were found to be less favourable under strongly acidic and strongly alkaline conditions. Under suitable conditions, for 50 mL industrial wastewaters (phenol: 100–160 mg/L; acetone: 680–800 mg/L), the amount of modified attapulgite was 1 g, the temperature was 80 °C and the pH was 7, and the maximum removal rates of acetone and phenol after 80 min reached about 65.71% and 78.72%, respectively, which were higher than those of ATP.

## 1. Introduction

Castor oil is a kind of triglyceride with natural fatty acids and is rich in China. The molecular structure of castor oil contains long hydrophobic fatty acid chain segments, unsaturated carbon–carbon double bonds, hydroxyl groups and ester-reactive groups [1]. It is the only hydroxyl-containing vegetable oil with an average hydroxyl functionality of 2.7 in its molecular structure [2]. As a non-edible oil, castor oil is also a natural, degradable and renewable resource [2]. Considering the environmental problems caused by non-renewable, biodegradable fossil fuels and scarce resources, there is a trend to replace fossil fuels with cheap and renewable vegetable oil resources. Castor oil is a widely used, cheap and renewable raw material, which is widely used for the production of surfactants, soaps, lubricants, coatings, dyes, plastics, pharmaceuticals, perfumes, etc. [3].

Quaternary ammonium salts refer to a class of substances that contain hydrophobic long alkyl chains and positively charged nitrogen atoms in their molecular structure [4]. Quaternary ammonium salts possess surface activity, adsorption and antibacterial activity. They are often used as emulsifiers for emulsion polymerisation [5,6], corrosion inhibitors [7], fabric softeners [8] and organic modifiers for clay mineral modification [9]. The surface properties of a natural sodium montmorillonite were drastically changed by dioctadecyl dimethyl ammonium chloride [10]. Sepiolite modified with tetradecyl trimethylammonium bromide, cetyl trimethylammonium bromide, and octadecyl trimethylammonium bromide showed a change in the surface wettability from strongly hydrophilic to increasingly hydrophobic, and a reversal of charge from negative to positive [11]. The commonly used quaternary ammonium salts are produced from petroleum feedstocks and have high toxicity. There is an urgent need to develop multifunctional quaternary ammonium salts with low toxicity and environmental friendliness.

Attapulgite is a natural clay mineral with silicate structures and is abundant in China. Due to its layered chain and fine regular structure, it has a multi-pore morphology and a large specific surface area, which gives it good adsorption performance, so it is widely used in the treatment of pollutants in water [12,13]. However, due to the hydrophilic surface of natural attapulgite, it has a weak adsorption capacity for organic pollutants in wastewater and often requires appropriate organic modification. An effective approach to improve the adsorption capacity and selectivity of attapulgite is the chemical surface modification of attapulgite with organic reagents [14]. Attapulgite modified with quaternary ammonium salt, which has an oil-friendly surface and can adsorb organic pollutants, has become an important issue in water pollution control. Huang and his co-author [15] investigated the selective adsorption of tannin on organically modified attapulgite clay with octadecyl trimethyl ammonium chloride (OTMAC). Cisneros-Rosado and co-workers used hexadecyl tributyl phosphonium bromide (HDTBP) to modify attapulgite, where the hydrophobic segments of HDTBP remain extended outward on the attapulgite surface, thus changing the hydrophilic nature of clay [16]. Attapulgite modified with dioctadecyl dimethylammonium bromide has significant implications for the development of adsorptive remediation materials for ionisable organic pollutants in wastewaters [17]. The removal rate of Acid Orange 7 and total organic carbon by attapulgite modified with cationic surfactant cetyltrimethylammonium bromide reached about 98.4% and 59.21%, respectively, after 2 h [18].

**Figure 1:**
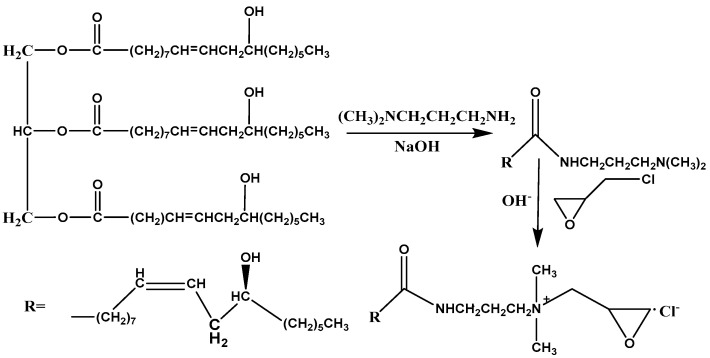
Synthesis route and chemical structure of castor oil-based quaternary ammonium salt.

In this study, castor oil, a renewable biomass, was used as a raw material and reacted with N,N-dimethyl-1,3-propanediamine to obtain castor oil amides, which were subsequently quaternised to obtain castor oil-based quaternary ammonium salts. The parameters such as reaction time, reaction temperature, molar ratio of raw materials and catalyst dosage were optimised. The synthesis route and chemical structure of castor oil-based quaternary ammonium salt are shown in Figure 1. Modified attapulgite was prepared using castor oil-based quaternary ammonium salt as a modifier. The structures of castor oil-based quaternary ammonium salt, modified attapulgite and attapulgite were compared by Fourier transform infrared spectra (FTIR) and X-ray diffraction (XRD) to speculate the modification mechanism. The surface microstructures of attapulgite before and after modification were observed by scanning electron microscopy (SEM). The removal rates of acetone and phenol from the industrial wastewaters were investigated by using modified attapulgite for wastewater treatment. Various factors such as the amount of modified attapulgite, temperature and pH were also investigated in batch experiments on the removal rates of acetone and phenol from wastewaters. The comparative experiments were conducted using modified attapulgite and attapulgite for wastewater treatment.

## 2. Materials and Methods

### 2.1. Materials

Analytical reagents such as N,N-dimethyl-1,3-propanediamine (DM) and epichlorohydrin (ECH) were purchased from Aladdin Reagent (Shanghai, China) Co., Ltd.; analytical reagent-grade methanol, sodium tetraphenyl boron, bromophenol blue and sodium hydroxide (NaOH) were purchased from Jiangsu Tongsheng Chemical Reagent Co., Ltd. Analytical castor oil (CO), n-hexane, ethanol, potassium bromide (KBr), cetyl trimethyl ammonium bromide (CTAB), hydrochloric acid (HCl) and other chemicals were purchased from Sinopharm Group Chemical Reagent Co., Ltd. (Shanghai, China). Attapulgite was supplied by Jiangsu Maige sorbent Co., Ltd. (Xuzhou, China). Attapulgite was washed, filtered, dried and ground at 30 °C to obtain pretreated attapulgite (ATP). Industrial wastewater samples (phenol: 100–160 mg/L; acetone: 680–800 mg/L; pH: 5–8) were taken from the actual wastewater of a chemical factory producing phenol acetone by the isopropyl benzene method; de-ionized water was prepared in our laboratory.

### 2.2. Synthesis

#### 2.2.1. Synthesis of Castor Oil-Based Quaternary Ammonium Salt (COQS)

As a general procedure, COQS synthesis was performed in two steps (see Figure 1). First, the desired amount of CO and NaOH dissolved in methanol (2.5 mL) solvent was added to a 250 mL three-neck flask equipped with a mechanical stirrer, reflux condenser and thermometer. DM was added dropwise at a drop rate of 0.25 mL/min. After the dropwise addition, the mixture was heated to a specified temperature for a period of time. The supernatant was repeatedly washed with deionised water to remove the lower turbid layer, concentrated with a rotary evaporator at 60 °C and then dried at 60 °C under vacuum to obtain a transparent yellowish liquid (castor oil-based amides). Secondly, an appropriate amount of castor oil-based amide was added to a 250 mL four-neck flask equipped with a mechanical stirrer, a reflux condenser, a nitrogen inlet and a thermometer. The reactor was degassed with nitrogen for 15 min before the reaction. When the temperature reached 48 °C, ECH (the molar ratio of amide to epichlorohydrin is 1:3) was added dropwise at a dropping rate of 0.25 mL/min. After the dropwise addition was completed, the mixture was heated at 50 °C for 2 h. The resulting product was repeatedly washed with hexane, purified and dried at 60 °C under vacuum to obtain a brown viscous liquid with 64.72% yield (COQS).

#### 2.2.2. Modification of Attapulgite

A certain amount of COQS was completely dissolved in 50 mL of water, and subsequently, ATP (5.0 g, dried at 105 °C) was dispersed in this solution. The suspension was subjected to ultrasound at 25 °C for a certain period of time. The modified ATP (M-ATP) was separated from the aqueous phase by filtration. M-ATP was washed with deionised water to remove excess quaternary ammonium salt molecules until no chloride ions were detectable in the filtrate by adding AgNO_3_ solution (0.1 mol/L) [19], then was dried at 80 °C under vacuum for 24 h, ground and sieved.

The grafting rate of the quaternary ammonium cationic salt on attapulgite was measured and calculated as follows [20]: 5 mL of the above M-ATP supernatant was added with 15 mL of distilled water and 0.5 mL of bromophenol blue indicator. This was then titrated with 0.02 mol/L sodium tetraphenyl boron until the blue colour faded to purple, and the grafting rate was calculated according to the following equation.

(1) $$Grafting\;rate\left(\%\right)=\left(1-\frac{0.02\times 50\times V_{1}}{5\times N}\right)\times 100\%$$

where $V_{1}$ is the volume of sodium tetraphenyl boron (mL) consumed by the titration; N is the amount of initial substance (mol) of the modifier in the treatment of attapulgite.

### 2.3. Characterisation

#### 2.3.1. COQS Characterisation

The infrared spectra of CO and COQS were recorded on a Nicolet iS 10 FT-IR measurement (Nicolet Co., Madison, WI, USA), and the samples were prepared with KBr salt film. The spectra were recorded over the range of 4000–500 cm^−1^, with a resolution of 4 cm^−1^. Different concentrations of COQS solutions were prepared, the pressure difference was measured by the maximum bubble method surface tension measurement device at 25 ± 0.1 °C and the surface tension of COQS solutions at different concentrations was determined by calculation.

#### 2.3.2. M-ATP Characterisation

ATP and M-ATP samples were prepared by KBr compression and characterised by FTIR; XRD analyses were performed on an X’Pert3Powder (PANalytical Co., Heracles Almelo, The Netherlands) with graphite filter slide, a tube pressure of 40 KV, a tube current of 40 mA, 2θ diffraction angle of 50–800 and scanning step of 0.026°/s; SEM characterisation was performed by a FEI QUANTA200 SEM (FEI Co., Hillsborough, OR, USA).

### 2.4. Application of M-ATP

Batch experiments were conducted to investigate the effects of pH, temperature and the amount of M-ATP on the removal rates of acetone and phenol in industrial wastewater. The pH of the industrial wastewater was adjusted from 4 to 10 by adding either diluted HCl or NaOH (0.1 mol/L). A series of 250 mL conical flasks containing 50 mL of industrial wastewater and various amount of M-ATP (0.2–1.4 g) were used at a certain temperature (25–90 °C) for 80 min, and the supernatant was taken for analysis after solid–liquid separation. The effects of experimental parameters such as pH, temperature of the solution and the amount of M-ATP were studied by varying one parameter and keeping the other parameters constant. Comparison experiments with ATP and M-ATP were performed as follows: two 50 mL untreated industrial wastewater samples were placed in a 250 mL conical flask, and 1 g of each ATP and M-ATP were added. The reaction was stirred at 80 °C for 80 min (150 rpm), and the supernatant was taken for analysis after solid–liquid separation. Phenol concentration was determined by TV-1810 UV–vis spectrophotometer (Beijing Puxi Universal Instrument Co., Ltd.). The uptake of phenol was monitored by measuring the absorbance at a λ_max_ of 265 nm, and its concentration was calculated according to the standard curves. The residual concentration of acetone in the supernatant was determined by a gas chromatographic method [21]. The acetone concentration was analysed by GC-1102 gas chromatography (Thermo Fisher Co., USA) with a flame ionisation detector. Based on the concentration change, the removal rates were calculated to evaluate the removal performance of ATP and M-ATP. Equation (2) shows how to calculate the removal (%) of phenol and acetone.

(2) $$emoval\;rate\left(\%\right)=\left(1-\frac{C_{1}}{C_{0}}\right)\times 100$$

where $C_{0}$ (mg/L) is the concentration of phenol or acetone in the initial wastewater; $C_{1}$ (mg/L) is the concentration of phenol or acetone in the treated wastewater.

## 3. Results and Discussion

### 3.1. COQS Synthesis Conditions

**Figure 2:**
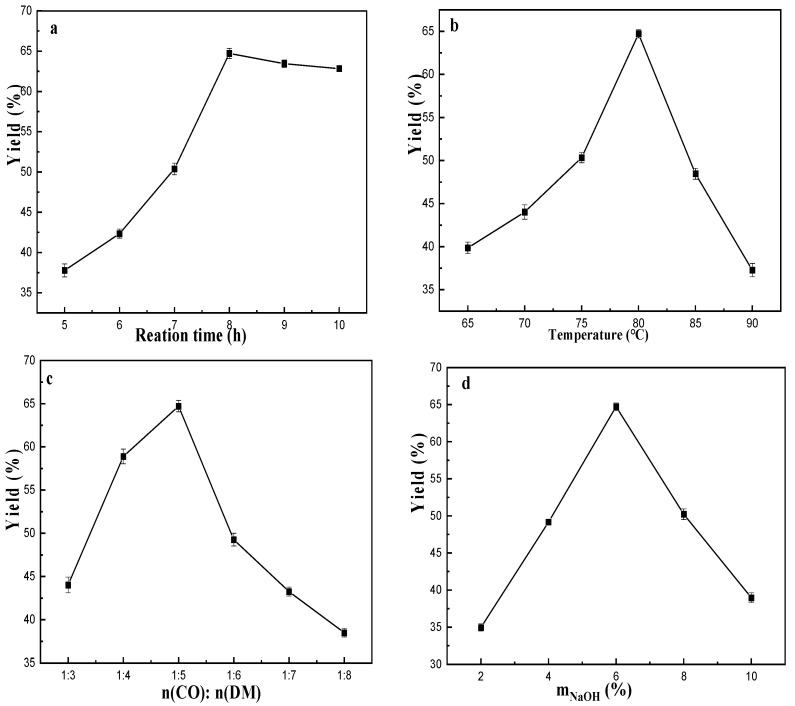
Effects of reaction time (**a**), reaction temperature (**b**), n(CO): n(DM) (**c**) and NaOH dosage (**d**) on the yield of COQS.

In order to improve the reaction efficiency and increase the yield of COQS, the parameters such as reaction time, reaction temperature, molar ratio of raw materials and catalyst dosage were optimised, and the results are shown in Figure 2.

It is well known that too short a reaction time leads to insufficient reaction and low yield; too long a reaction time increases the production cost. The COQS yield increased linearly with the increase of reaction time from 5 to 8 h (Figure 2a). The yield reached a maximum of approximately 64.72% at 8 h. Within 8–10 h, the yield curve flattened with increasing reaction time. As shown in Figure 2b, the acylation rate accelerated, and the COQS yield increased with the gradual increase of reaction temperature within 65–80 °C. The yield reached the highest value at 80 °C. It is well known that amides hydrolyse easily under alkaline conditions at high temperatures. Above 80 °C, the COQS yield decreased with increasing temperature; high reaction temperatures may have led to hydrolysis and other side reactions of castor oil-based amides, resulting in lower yields. When n(CO): n(DM) was in the range of 1:3–1:5, COQS yield showed an increasing trend with the increase of n(DM) (Figure 2c), and when n(CO): n(DM) was in the range of 1:5–1:8, too much DM will interfere with the catalytic performance of the catalyst, leading to a significant decrease in COQS yield. Therefore, the optimum molar ratio is 1:5. The catalyst dosage within a certain range will affect the reaction rate and product yield. The effect of catalyst dosage on yield was discussed separately in the range of 2–10% NaOH (mass percent of castor oil), and the results are shown in Figure 2d. After increasing the catalyst dosage, the COQS yield increased significantly to a maximum of approximately 64.72% and decreased thereafter, probably because the excessive catalyst dosage led to other side reactions that affected the COQS yield.

### 3.2. Organic Modification Parameters of ATP

COQS was used to modify ATP by ultrasound. In this section, the process conditions for the modification of ATP were studied using the grafting rate as an indicator. The effects of COQS addition amount, ultrasonication time and pH on grafting rate were investigated.

**Figure 3:**
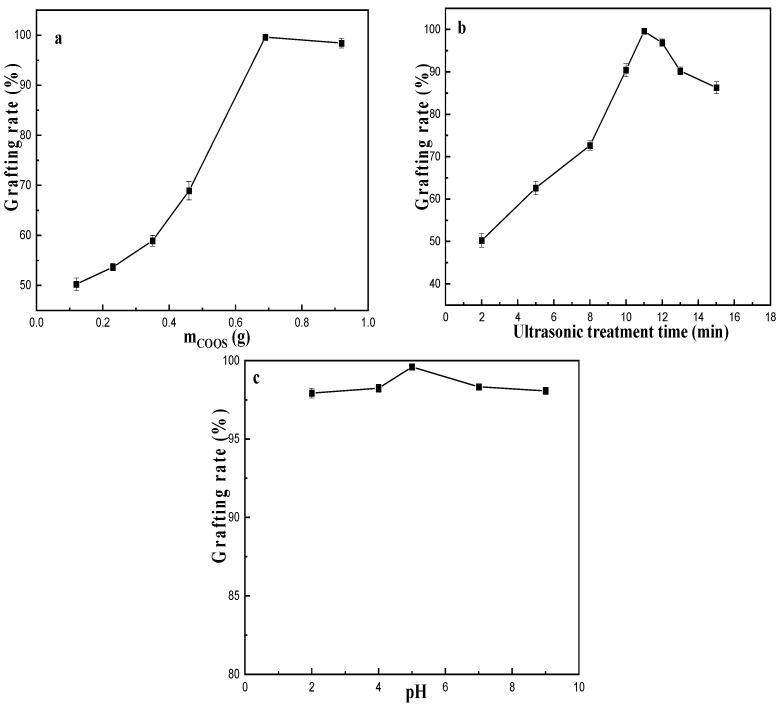
Effects of the amounts of COQS (**a**), ultrasound treatment time (**b**) and pH (**c**) on the grafting yield.

As shown in Figure 3a, when COQS addition amount was 0.1–0.69 g, the grafting rate increased linearly with the increase of COQS; when COQS was added at 0.69–0.92 g, the grafting rate on attapulgite changed very little. Surface tension measurements showed COQS to be surface active and slightly foamy. A large amount of addition is not only unfavourable for washing M-ATP, but also leads to agglomeration of the modifiers, which may result in the failure of the modification [22]. Therefore, 0.69 g of quaternary ammonium salt was added per 5 g of ATP. Within 2–11 min, the grafting rate increased with increasing ultrasound treatment time (Figure 3b), and the highest grafting rate reached about 99.6% when the ultrasound treatment time was 11 min. The grafting rate decreased slightly when the ultrasound treatment time continued to be extended. This may be due to the fact that the ultrasound treatment promoted the interaction between attapulgite and COQS within 2–11 min, and when the ultrasound treatment time was continued to be prolonged, the grafting rate decreased since the strong force of the ultrasound waves destroyed the grafted COQS. From Figure 3c, the maximum grafting rate was about 99.6% when the pH was 5. The grafting rate changed very little when the pH was varied from 2 to 9, indicating that the pH of the solution had little effect on the grafting effect of COQS on attapulgite. The ion exchange properties of clay particles are strongly dependent on pH [23]. Therefore, it was speculated that the interaction between the quaternary ammonium cations and attapulgite did not result from an ion-exchange interaction with the cations between attapulgite fibre crystals.

### 3.3. Characterisation

#### 3.3.1. COQS Characterisation

**Figure 4:**
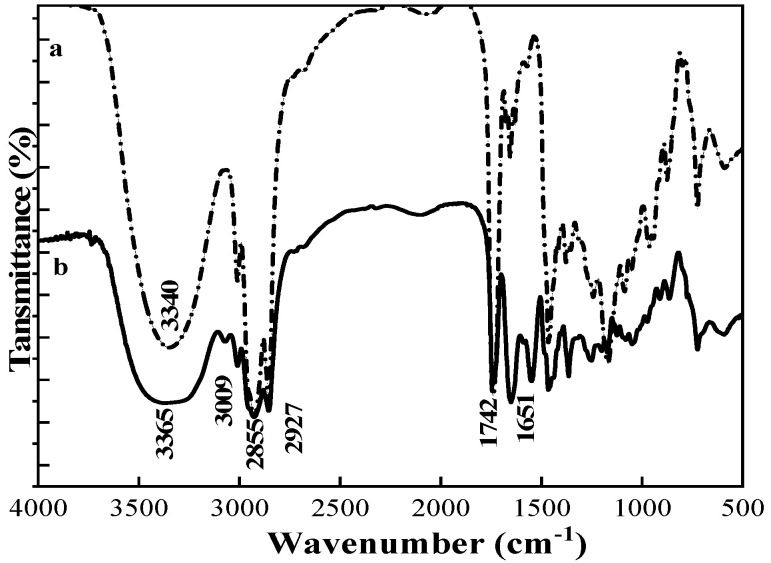
FTIR spectra of CO (**a**) and COQS (**b**).

Figure 4 shows the infrared spectra of CO and COQS. There is a hydroxyl absorption peak at 3340 cm^−1^ for CO. Compared with the spectrum of CO, the broad peak at 3365 cm^−1^ in the infrared spectrum of COQS is the N-H stretching vibration, and the peaks at 3009 cm^−1^, 2927 cm^−1^ and 2855 cm^−1^ can be attributed to the C-H stretching vibration absorption peaks of methylene and methyl groups with stronger absorption peaks, reflecting the structural characteristics of the trialkylmethyl salt. The stretching vibration of C=O of the α-unsaturated ester with conjugated structure is at 1742 cm^−1^. The absorption peak at 1651 cm^−1^ is the characteristic absorption peak of the stretching vibration of the C=C bond.

**Figure 5:**
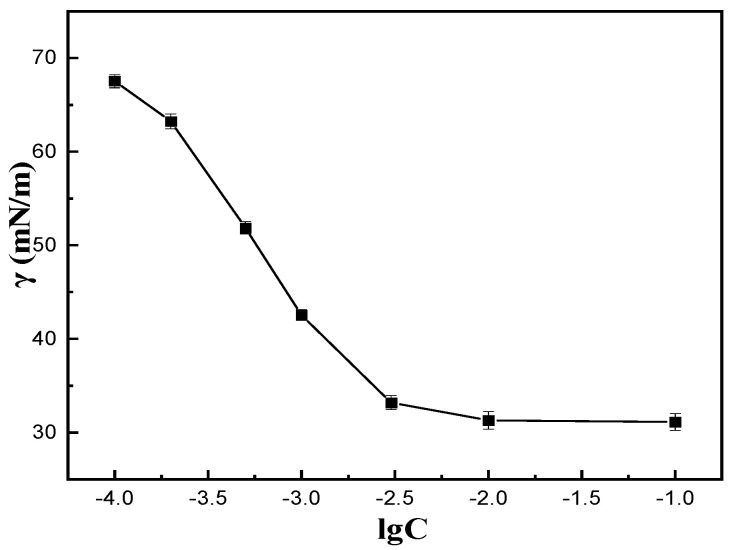
γ−lgC curve of COQS.

The critical micelle concentration was determined from the surface tension (γ) and the log molar concentration (C) curve of the surfactant at 25 °C. As can be seen from Figure 5, the surface tension decreased rapidly at first with the increase of COQS molar concentration. When lgC was close to −2.0, the surface tension did not change significantly. At this time, the surface tension was 31.21 mN/m, and the critical micelle concentration was 2.51 × 10^−3^ mol/L, which was of the same order as the critical micelle concentration (1.09 × 10^−3^ mol/L) of CTAB in the literature [24]. This result indicates that the synthesised COQS have some surface activity.

#### 3.3.2. M-ATP Characterisation

The structures and properties of the modified products were characterised, and the modification mechanism will be inferred.

**Figure 6:**
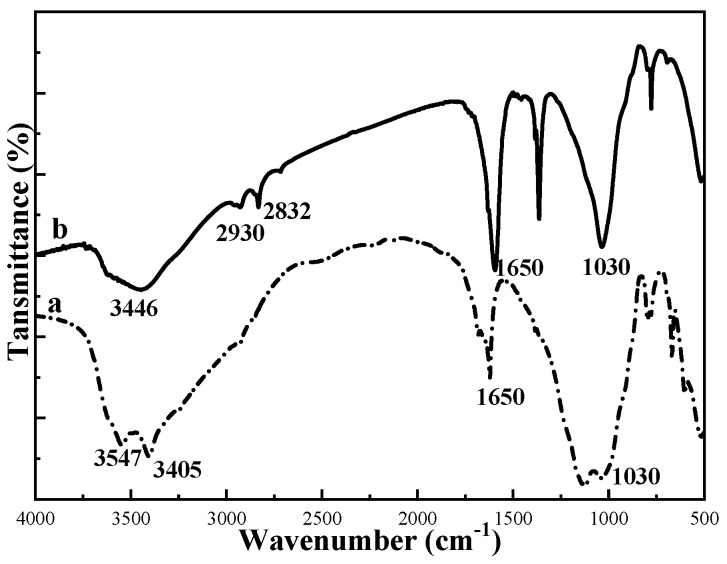
FTIR spectra of ATP (**a**) and M-ATP (**b**).

The FTIR spectra of ATP and M-ATP are shown in Figure 6. The broad adsorption peaks of ATP are at 3547 cm^−1^, 3405 cm^−1^ and 1650 cm^−1^, corresponding to the coordination water, adsorbed water and bound water, respectively [25]. The peak at 1030 cm^−1^ is associated with the stretching vibration of the Si-O-Si bond [26]. Unlike ATP, the wide absorption peak of M-ATP at 3446 cm^−1^ is the N-H stretching vibration; the absorption peaks at 2930 cm^−1^ and 2832 cm^−1^ are the C-H stretching vibrations of methyl and methylene groups in the long alkyl chains of COQS. The results indicate that organic functional groups were adsorbed on the surface of attapulgite treated with COQS.

**Figure 7:**
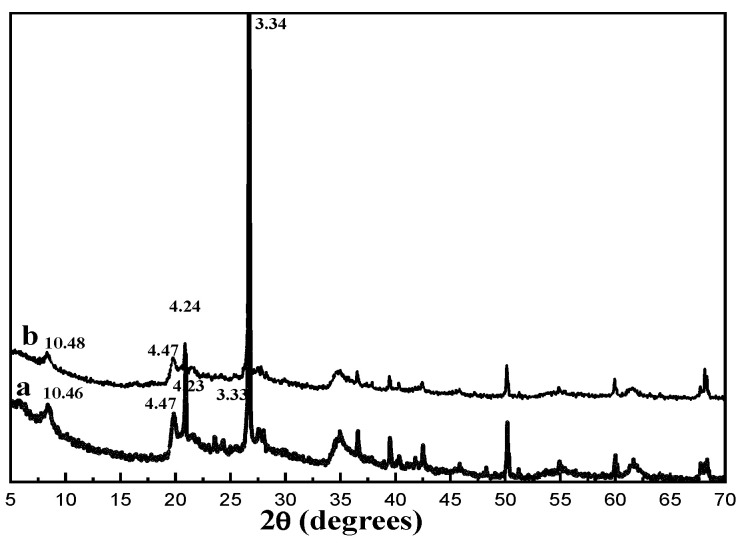
XRD spectra of ATP (**a**) and M-ATP (**b**).

As can be seen in Figure 7, the spectra in both a and b showed the characteristic diffraction peaks of the samples at 2θ = 8.4, 29.8, 26.6 and 35.3; these correspond to the (110), (040), (400) and (440) crystal faces of attapulgite, respectively [27]. Both samples exhibit a crystalline character. The characteristic spacings of ATP were 10.46 Å, 4.47 Å, 4.23 Å and 3.33 Å, and those of M-ATP were 10.48 Å, 4.47 Å, 4.24 Å and 3.34 Å, respectively. The characteristic spacings of M-ATP do not change significantly compared to those of ATP. This shows that the crystal structure of M-ATP remains unchanged and the surfactant is only bound to the surface of ATP, without inserting into the nanochannels of the ATP [14,28]. This is similar to the results of Li [11], who used CTAB to modify natural sepiolite and found that the crystal structures of CTAB-modified sepiolite were identical to those of the original sepiolite, and the d-values of CTAB-modified sepiolite were almost constant to those of the original sepiolite. They explained that the CTAB surfactant molecules were mainly covered on the outer surface of the clay particles and/or loaded at the edges of the clay particles.

**Figure 8:**
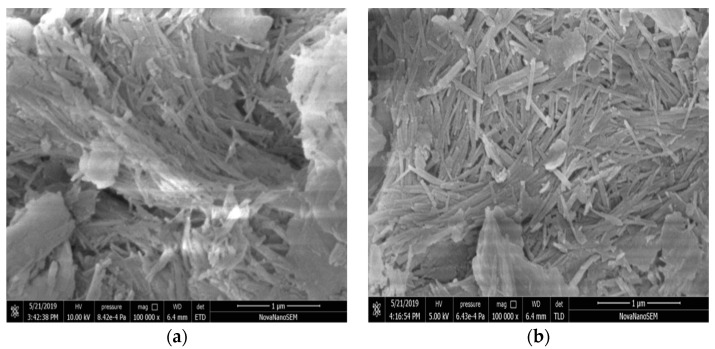
SEM images of ATP (**a**) and M-ATP (**b**).

The SEM images of ATP and M-ATP are shown in Figure 8. ATP exhibits a densely ordered fibrous structure consisting of closely and parallel arranged single crystals which assemble into crystal bundles and form particles. In contrast, most of the rod crystal bundles of M-ATP were separated into single rod crystals, and the crystal bundles were obviously dispersed. The microstructure consisting of fibres was relatively loose and fibrous. This might be due to the presence of the organic substance, COQS, on the surface of the rod crystals or bundles, which weakened the original intermolecular binding force and reduced the affinity among them, leading to the reduction of bundle agglomeration [16]. These results are in agreement with those of Peng [29].

### 3.4. Application of M-ATP

**Figure 9:**
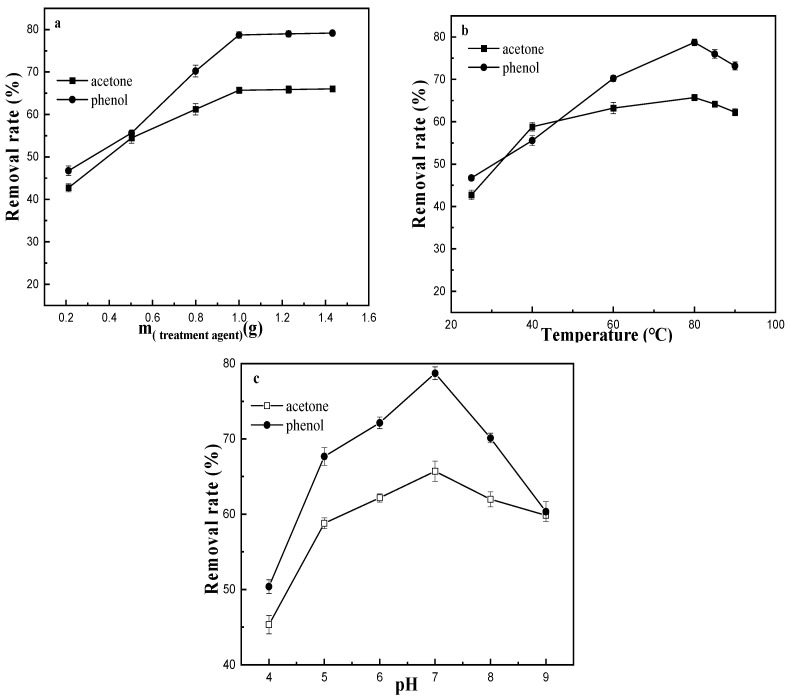
Effects of the amounts of M-ATP (**a**), treatment temperature (**b**) and pH (**c**) on the removal rate.

The effects of the amount of M-ATP (a), treatment temperature (b) and pH (c) on the removal rate are shown in Figure 9. As the amount of M-ATP increased, the removal rates of acetone and phenol increased linearly and then levelled off. This indicated that the best removal effect was achieved when the amount of M-ATP is 1g. Temperature is the major parameter affecting the adsorption properties [11]. Figure 9b clearly reveals that the removal rate increased rapidly with increasing temperature in the range of 25–80 °C. Above 80 °C, the removal rate decreased slowly. The effect of temperature influences the adsorption capacity by affecting the molecular interactions and solubility [30,31]. When the temperature of the system was increased, the phenol or acetone molecules are more active, which increases the contact between M-ATP and the phenol or acetone molecules, thus enhancing the adsorption capacity. However, if the temperature is too high, this may lead to a decrease in the interaction force between the quaternary ammonium cation and ATP, reducing the removal rates. pH is one of the most important parameters in the adsorption process because it affects the physicochemical properties of the surface and the surface binding sites of the adsorbents [32]. In this study, the initial pH of the industrial wastewater samples was 5–8, so the pH was adjusted from 4 to 10. The removal rates increased in the pH range of 4–7 and decreased with further increases in pH. This can be explained by the change in the number of protons in the solution. At a lower pH (pH < 7), more protons were available and saturated the M-ATP sites, increasing the cationic properties of the M-ATP surface, which greatly reduced the hydrophobic properties of M-ATP and hindered further removal of acetone and phenol. The increased removal rates at a relatively high pH were due to the lower number of protons, which preserved the hydrophobicity of the M-ATP surface [32]. The removal rates decreased rapidly with the increase of hydroxyl ions in the solution above a pH of 8, indicating that high pH was not conducive to the removal of acetone and phenol because excess hydroxyl ions in the solution compete with acetone or phenol for active M-ATP sites [33]. Removal was found to be less favourable under strongly acidic conditions, and the maximum removal capacity was observed at about neutrality.

**Figure 10:**
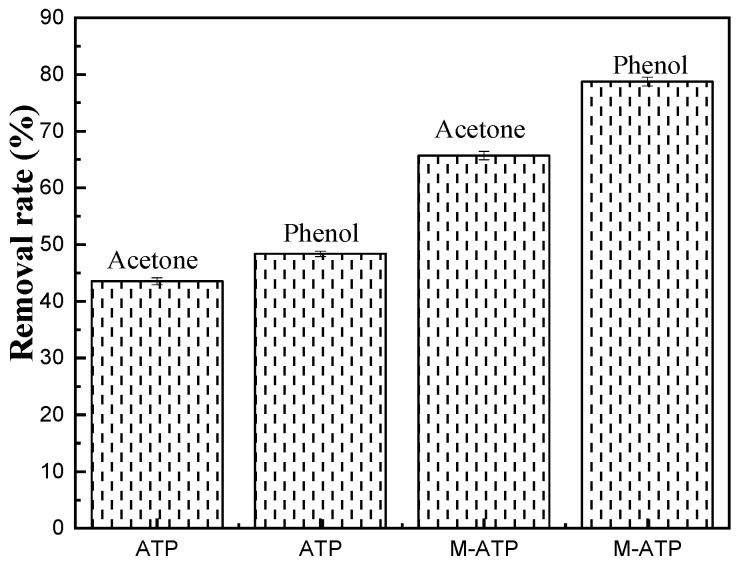
Removal rates of ATP and M-ATP.

The removal rates of acetone and phenol were about 43.55% and 48.39%, respectively, for ATP (Figure 10). The removal rates of acetone and phenol by M-ATP were about 65.71% and 78.72%, respectively, which were higher than those by ATP. According to reports in the literature [34], the hydrophobic segments of COQS extended outward to modify the hydrophilic nature of ATP. The organic modifier with a longer carbon chain would exhibit better hydrophobicity [35]. The adsorption of acetone and phenol by M-ATP was significantly improved, which may be due to the fact that the quaternary ammonium salt, as an organic modifier, made the originally hydrophilic attapulgite surface hydrophobic and lipophilic.

## 4. Conclusions

In this study, a green and efficient production process for castor oil derivatives was developed, which is of theoretical importance and practical application for the development of quaternary ammonium salts with different molecular structures and the exploitation of their advantages as modifiers in wastewater treatment.

The synthesis conditions for the castor oil-based quaternary ammonium salt were as follows: reaction temperature of 80 °C, reaction time of 8 h, n(CO): n(DM) of 1:5, catalyst 6% NaOH and product yield of about 64.72%. The grafting rate of COQS on ATP was about 99.6% when the amount of modifier was 0.69 g per 5 g of ATP, the ultrasound treatment time was 11 min and the pH was 5. COQS successfully modified the ATP. The crystal structure of M-ATP remains unchanged and the surfactant is only bound to the surface of ATP without inserting into the nanochannels of ATP. Most of the rod crystal bundles of M-ATP were split into single rod crystals, and the crystal bundles were obviously dispersed. The microstructure consisting of fibres was relatively loose and fibrous. The synthesised castor oil-based quaternary ammonium salt can be used as a modifier of attapulgite, which is efficiently bound to the surface of attapulgite.

The removal rates depended on the amount of M-ATP, pH and temperature. Removal rates were less favourable under strongly acidic and strongly alkaline conditions, with maximum removal rates observed under near-neutral conditions. The removal rates of acetone and phenol increased linearly with increasing amounts of M-ATP and then levelled off. The maximum removal rates were obtained at 50 mL of industrial wastewater (phenol: 100–160 mg/L; acetone: 680–800 mg/L), 1 g of modified attapulgite, a temperature of 80 °C and a pH of 7. The removal rates of acetone and phenol by M-ATP were about 65.71% and 78.72%, respectively, which were higher than those by ATP. This confirmed the potential of attapulgite modified by castor oil-based quaternary ammonium salt for the effective treatment of wastewater.

## Data Availability

The data are available on request from the corresponding author.
